# Cost-effectiveness of sacituzumab govitecan versus single-agent chemotherapy for metastatic triple-negative breast cancer: a trial-based analysis

**DOI:** 10.1186/s12962-024-00539-y

**Published:** 2024-04-24

**Authors:** Yilai Wu, Shanshan Hu, Xiaolin Liu, Yang Chen, Jiajie Luan, Shuowen Wang

**Affiliations:** 1 Department of Clinical Pharmacy, The First Affiliated Hospital of Wannan Medical College, 241001 Wuhu, Anhui China; 2 Department of Clinical Pharmacy, Shanghai General Hospital, Shanghai Jiao Tong University School of Medicine, 200080 Shanghai, China

**Keywords:** Cost-effectiveness analysis, Sacituzumab govitecan, Single-agent chemotherapy, Metastatic triple-negative breast cancer

## Abstract

**Background:**

Sacituzumab govitecan (SG) has recently been approved in China for the post-line treatment of metastatic triple-negative breast cancer (mTNBC). SG substantially improves progression-free survival and overall survival compared with single-agent chemotherapy for pretreated mTNBC. However, in view of the high price of SG, it is necessary to consider its value in terms of costs and outcomes. This study aimed to estimate the cost-effectiveness of SG versus single-agent treatment of physician’s choice (TPC) in the post-line setting for patients with mTNBC from a Chinese healthcare system perspective.

**Methods:**

The cohort characteristics were sourced from the ASCENT randomized clinical trial, which enrolled 468 heavily pretreated patients with mTNBC between November 2017 and September 2019. A partitioned survival model was constructed to assess the long-term costs and effectiveness of SG versus TPC in the post-line treatment of mTNBC. Quality-adjusted life-months (QALMs) and total costs in 2022 US dollars were used to derive incremental cost effectiveness ratio (ICER). QALMs and costs were discounted at 5% annually. The willingness-to-pay (WTP) threshold was defined as $3188 per QALM, three times China’s average monthly per capita gross domestic product in 2022. One-way sensitivity analysis, probabilistic sensitivity analysis, and scenario analyses were performed to estimate the robustness of the results.

**Results:**

Treatment with SG yielded an incremental 5.17 QALMs at a cost of $44,792 per QALM, much above the WTP threshold of $3188/QALM in China. One-way sensitivity analysis showed that SG price was a crucial factor in the ICER. Probabilistic sensitivity analysis revealed that the cost-effective acceptability of SG was 0% in the current setting. Scenario analyses indicated that the result was robust in all subgroups in ASCENT or under different time horizons. Furthermore, SG must reduce the price to enter the Chinese mainland market. When the monthly cost of SG reduce to $2298, SG has about 50% probability to be a preferred choice than TPC.

**Conclusions:**

SG was estimated to be not cost-effective compared with TPC for post-line treatment for mTNBC in China by the current price in HK under a WTP threshold of $3188 per QALM. A drastic price reduction is necessary to improve its cost-effectiveness.

**Supplementary Information:**

The online version contains supplementary material available at 10.1186/s12962-024-00539-y.

## Background

Breast cancer is the most common cancer in women with 2.3 million new cases diagnosed and 685,000 deaths worldwide in 2020 [1]. Triple-negative breast cancer which accounts for 15% of breast cancer cases is defined as lacking expression of estrogen receptor, progesterone receptor and human epidermal growth factor receptor type 2 (HER2) [2]. Endocrine therapy or HER2-targeted therapy are totally ineffective for patients with triple-negative breast cancer. Some potential therapies are still in the laboratory stage [3]. Currently, chemotherapy remains the mainstay of systemic therapy, especially for previously treated metastatic triple-negative breast cancer (mTNBC) [4]. However, chemotherapy is associated with a low response rate and short progression-free survival [5]. There is an urgent need for new treatment options to improve therapeutic outcomes.

Sacituzumab govitecan (SG) is an antibody–drug conjugate consisting of a trophoblast cell-surface antigen 2 (Trop-2) targeting antibody coupled to a topoisomerase I inhibitor SN-38 by a proprietary hydrolysable linker [6]. The ASCENT randomized clinical trial compared SG with single-agent treatment of physician’s choice (TPC) for previously treated mTNBC [7]. Four representative single-agent chemotherapy regimens: eribulin, vinorelbine, capecitabine, and gemcitabine were offered for selection in the TPC arm. Patients who received SG showed a substantial survival benefit compared with those who received TPC with respect to progression-free survival (hazard ratio [HR], 0.41; *p* < 0.001) and overall survival (HR, 0.48; *p* < 0.001). The percentage of patients with an objective response was higher with SG than TPC (35% vs. 5%). The benefit with SG was observed in all prespecified subgroups. Nevertheless, adverse events (AEs) were more frequent with SG, particularly myelosuppression and diarrhea. Fortunately, these AEs are generally manageable, leading to a 5% incidence of treatment discontinuation. SG was subsequently approved in the Chinese mainland in June 2022 to treat recurrent or refractory TNBC.

However, as SG has not yet been priced or marketed in Chinese mainland, patients who need to use it generally have to purchase it from Hong Kong. Therefore, there is an impetus to evaluate the cost-effectiveness of SG at the current Hong Kong price in the Chinese setting to provide a reference for medical decision-making and insurance reimbursement. In addition, an initial exploration of future pricing in Chinese mainland is warranted. This study was thus conducted to evaluate the cost-effectiveness of SG vs. TPC in the post-line setting for mTNBC from the perspective of the Chinese healthcare system.

## Methods

### Model structure

**Fig. 1:**
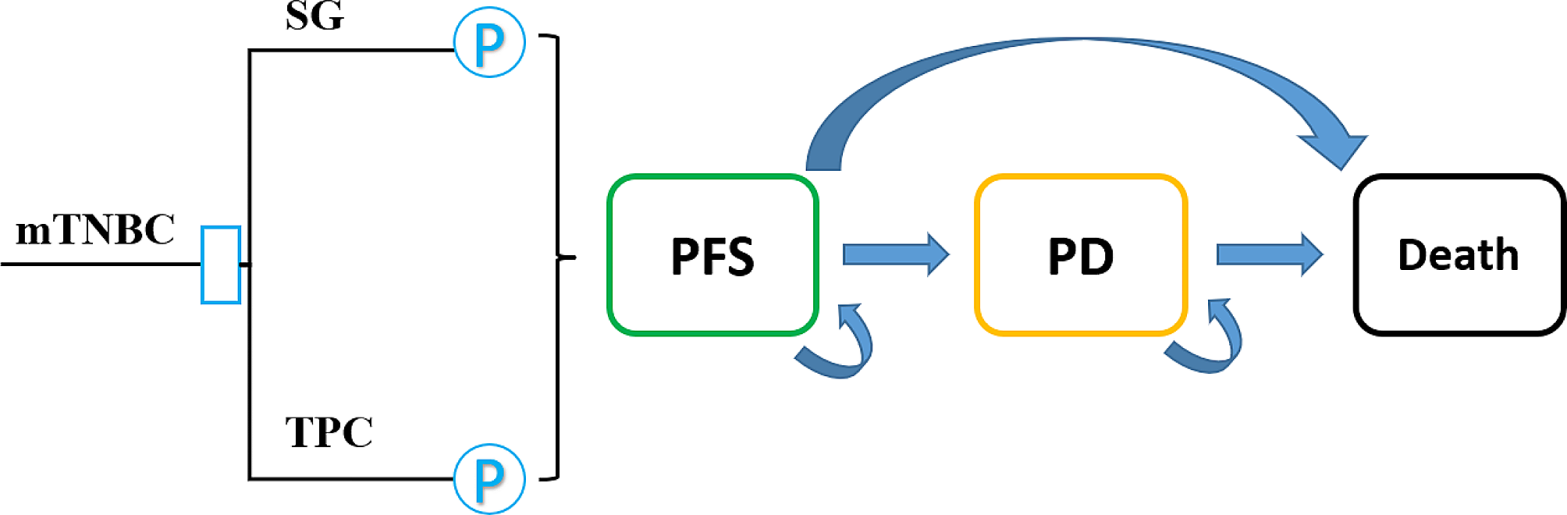
Partitioned survival model diagram. (Note: mTNBC, metastatic triple-negative breast cancer; SG, sacituzumab govitecan; TPC, treatment of physician’s choice; PFS, progression-free survival; PD, progressed disease; P, partitioned survival analysis)

A partitioned survival model (PSM) was developed to estimate the costs and long-term outcomes of SG and TPC in mTNBC. Eribulin, vinorelbine, capecitabine, and gemcitabine were included in the TPC group. The disease process was simulated as three states: progression-free survival (PFS), progressed disease (PD) and death states. The model structure is shown in Fig. 1. All patients were assumed to be in the PFS state at the beginning of simulation, all treatments were continued until disease progression or unacceptable AEs, and both groups received best supportive care (BSC) after disease progression until death. To be consistent with the survival reporting unit, the cycle length in the model was set to 1 month. The time horizon was determined to be 10 years to adequately reflect the survival of patients with mTNBC. An annual discount rate of 5% was adopted for both costs and outcomes, as recommended in China [8]. The primary outputs of the model were total costs, quality-adjusted life months (QALMs), incremental cost-effectiveness ratio (ICER), net monetary benefit (NMB), and incremental net monetary benefit (INMB). The PSM was generated by TreeAge Pro 2022 (TreeAge Software, Williamstown, Massachusetts, USA).

### Survival analysis

**Fig. 2:**
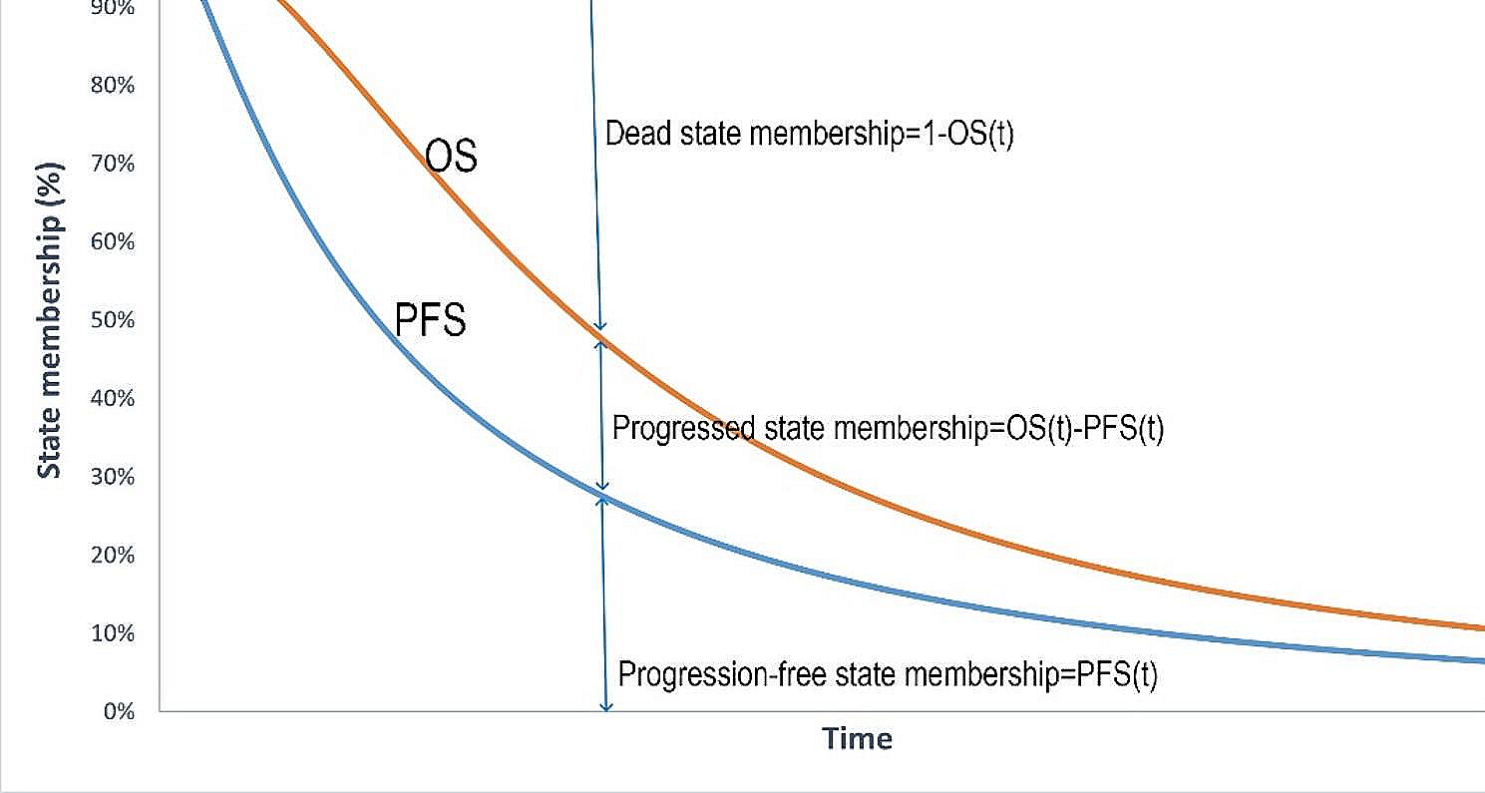
State membership in a 3-state partitioned survival model. (Note: OS, overall survival; PFS, progression-free survival)

Cohort characteristics were sourced from the ASCENT randomized clinical trial, which enrolled 468 taxane-pretreated patients with mTNBC between November 2017 and September 2019. As the individual patient data (IPD) of ASCENT trial were not accessible, the reconstructed data of OS and PFS were obtained from the survival curves reported in the ASCENT trial at first using GetData Graph Digitizer software (version 2.24, GetData Pty Ltd., Kogarah, Australia). Next, IPD and Kaplan-Meier curves during the follow-up period were reconstructed according to the algorithm developed by Guyot [9]. Data processing and analyses were conducted by R software (version 4.2.2, R Foundation for Statistical Computing, Vienna, Austria). The reconstructed Kaplan-Meier curves are shown in Supplementary Fig.S1 and Fig.S2. Finally, the optimal fitting models were selected from exponential, gamma, Gompertz, Weibull, log-logistic and log-normal by Akaike’s information criterion (AIC) and Bayesian information criterion (BIC). The results of model fitting are shown in Supplementary Fig.S3 and Supplementary Tab.S1. The parameters of the best-fitted models were obtained to describe the OS and PFS profile beyond the follow-up period. In the three-state PSM, OS and PFS curves were used to estimate state membership at a certain time point. The PFS state membership was directly provided by the area under the PFS curve. As the area under the OS curve was represented as the proportion of live patients, the dead state membership was simply 1 minus the OS curve at each time point. For the PD state, membership was derived as the difference between the OS and the PFS curve at each time point, as this provided the proportion of patients who are alive but not progression-free [10]. These states’ membership were illustrated in Fig. 2.

### Utility estimates

The quality-adjusted life expectancy was calculated by combining survival time and utility. Utilities of 0.86 and 0.73 were used to describe the quality of life (QoL) of Chinese breast cancer patients in chemotherapy and relapse, respectively, as previously published [11]. In the ASCENT trial, the European Organization for Research and Treatment of Cancer Quality of Life of Cancer Patients (EORTC QLQ-C30) questionnaire was adopted to evaluate the QoL benefits of different treatments. The results demonstrated that the SG group had consistently higher EORTC QLQ-C30 scores than the TPC group [12]. To account for the improvement in QoL, the utility increment was estimated by mapping the EORTC QLQ-C30 to the EQ-5D using the algorithm derived by Gray [13]. According to the calculation, the utilities of the SG and TPC groups were respectively 0.679 and 0.659 at baseline and were assessed every 4 weeks. After 6 cycles of treatment, the SG group had a mean utility value of 0.711, and the TPC group had a mean value of 0.644. This means that SG improved the QoL of mTNBC patients by 0.032 utility values, whereas TPC decreased QoL by 0.015. Therefore, the utility increment was approximately 0.047. The parameters involved in the calculation are listed in Supplementary Tab.S2 The utility increment was then added to the utility value for chemotherapy (0.860) to estimate that for SG (0.907).

### Cost estimates

This study was based on the perspective of the Chinese healthcare system and should have included all healthcare-related costs. Because of the difficulty in measuring indirect medical costs, the analysis included only direct medical costs. In the PFS state, costs included costs of TPC or SG drug acquisition, administration, treatment of AEs, and follow-up. In the PD state, costs included costs of BSC and follow-up. In the death state, costs merely included costs of terminal care, which expressed as the exit cost of PD in model. The baseline age of patients with mTNBC in the ASCENT trial was 54 years, and the average weight and height of Chinese women at this age were 157.2 cm and 60.8 kg, respectively [14]. The body surface area was calculated to be 1.58m^2^. The doses and costs of drugs were calculated based on the above data and are shown in Supplementary Tab.S3. To simplify the model, AEs with a grade ≥ 3 while rate ≥ 5 reported in the ASCENT trial were included (Supplementary Tab.S4). It is important to note that the occurrence of grade 3–4 AEs means that the current regimen needs to be changed, the costs of AEs were thus calculated only once in each regimen. Other key cost parameters retrieved from the database and published literature are shown in Table 1. The Chinese RMB was converted into US dollars using the average exchange rate in 2022 (1 USD = 6.7208 Yuan). The Hong Kong price of SG was adopted and converted at 1 USD = 7.8305 HKD. All costs were inflated to 2022 values based on the Chinese Consumer Price Index (CPI) for healthcare [15]. The willingness-to-pay (WTP) threshold was set at $3188/QALM, which is 3 times the Chinese average monthly GDP per capita in 2022, to assess cost-effectiveness in China [16].

**Table 1:** Model parameters: baseline values, ranges and distributions for sensitivity analysis

Model parameters	Baseline	Minimum		Maximum	Distribution	Reference	
Weibull survival model for OS of SG	Shape = 1.4178, Scale = 15.6718					[7]	
Log-logistic survival model for OS of TPC	Shape = 1.895, Scale = 6.578					[7]	
Log-normal survival model for PFS of SG	Meanlog = 1.5362, Sdlog = 1.0136					[7]	
Log-logistic survival model for PFS of TPC	Shape = 2.325, Scale = 2.135					[7]	
**Risk for main AEs in SG**							
Neutropenia	0.51	0.41		0.61	Beta	[7]	
Anemia	0.08	0.06		0.10	Beta	[7]	
Leukopenia	0.10	0.08		0.12	Beta	[7]	
Febrile neutropenia	0.06	0.05		0.07	Beta	[7]	
Diarrhea	0.10	0.08		0.12	Beta	[7]	
**Risk for main AEs in chemotherapy**							
Neutropenia	0.33	0.26		0.40	Beta	[7]	
Anemia	0.05	0.04		0.06	Beta	[7]	
Leukopenia	0.05	0.04		0.06	Beta	[7]	
Fatigue	0.05	0.04		0.06	Beta	[7]	
**Proportion of each regimen in TPC group**							
Eribulin	0.54	0.43		0.65	Beta	[7]	
Vinorelbine	0.20	0.16		0.24	Beta	[7]	
Capecitabine	0.13	0.10		0.16	Beta	[7]	
Gemcitabine	0.12	0.10		0.14	Beta	[7]	
**Utility**							
Chemotherapy	0.860	0.790		0.920	Beta	[11]	
SG	0.907	0.820		0.960	Beta	[11]	
PD	0.730	0.690		0.760	Beta	[11]	
**Drug cost, $ per month**							
SG	33,277	26,622		39,932	Gamma	[17]	
Eribulin	679	543		815	Gamma	[18]	
Vinorelbine	714	571		857	Gamma	[18]	
Capecitabine	414	331		497	Gamma	[18]	
Gemcitabine	242	194		290	Gamma	[18]	
**Cost of AEs, $ per event**							
Neutropenia	740	592		888	Gamma	[19]	
Anemia	1566	1253		1879	Gamma	[20]	
Leukopenia	740	592		888	Gamma	[19]	
Febrile neutropenia	766	613		919	Gamma	[20]	
Diarrhea	743	594		892	Gamma	[20]	
Fatigue	165	132		198	Gamma	[20]	
**Other costs, $ per event**							
Follow up	775	620		930	Gamma	[21]	
Administration	21	17		25	Gamma	Local charge	
BSC	167	134		200	Gamma	[22]	
Terminal care	1981	1585		2377	Gamma	[22]	

Note: SG, sacituzumab govitecan; PFS, progression-free survival; PD, progressed disease; AEs, adverse events; BSC, best supportive care

### Sensitivity analyses

One-way sensitivity analysis and probabilistic sensitivity analysis were conducted to explore the robustness of the base-case results. In one-way sensitivity analysis, the included parameters varied over a credible range that was obtained from 95% credible intervals or by assuming a 20% variance from the base-case values [23, 24]. A total of 1000 Monte Carlo simulations were performed to conduct a probabilistic sensitivity analysis in which the parameters were varied simultaneously with a prespecified distribution. All parameters, including baseline values, ranges, and distributions in the sensitivity analyses are shown in Table 1.

### Scenario analyses

To estimate the indeterminacy of cost-effectiveness in different subpopulations, cost-effectiveness analyses were conducted for the subgroups presented in the ASCENT trial by varying the HRs for PFS. In addition, 1 year, 3 years, 5 years, and 10 years (base-case) of time horizon were respectively performed in PSM to simulate real-world survival scenarios. Finally, to provide a reference for future drug pricing, we also explored the threshold price that makes SG just cost-effective than TPC for mTNBC patients in Chinese mainland. The cost-effectiveness analyses report follows the Consolidated Health Economic Evaluation Reporting Standards (CHEERS) reporting guidelines [25] (Supplementary Tab.S6).

## Results

### Base-case analysis

According to the model outputs, patients who received SG gained 12.29 QALMs, that was 5.17 QALMs more than patients who received TPC. However, the SG regimen incurred an additional cost of $231,378 compared with TPC, resulting in an ICER of $44,792 per QALM which is much higher than the WTP threshold of $3188/QALM in Chinese mainland (Table 2). These results suggest that SG is not a cost-effective therapeutic regimen for mTNBC than single-agent chemotherapy in Chinese mainland at its current price in Hong Kong.

**Table 2:** Cost and outcome results in base-case analysis

Strategy	SG	TPC	SG vs. TPC
Cost, $			
Cost of progression-free state	233,023	2251	230,772
Cost of post-progression state	4797	4191	606
Total cost	237,821	6442	231,378
QALM, month			
QALM of progression-free state	7.19	3.19	3.99
QALM of post-progression state	5.10	3.93	1.17
Total QALM	12.29	7.12	5.17
ICER*, $/QALM	-	-	44,792
NMB	-198,641	16,269	-
INMB	-	-	-214,910

*Compared to TPC at a willing-to-pay of $3188/QALM in China.

Note: SG, Sacituzumab govitecan; TPC, single-agent treatment of physician’s choice; ICER, incremental cost-effectiveness ratio; QALM, quality adjusted life month; NMB, net monetary benefit; INMB, incremental net monetary benefit.

### Sensitivity analyses

**Fig. 3:**
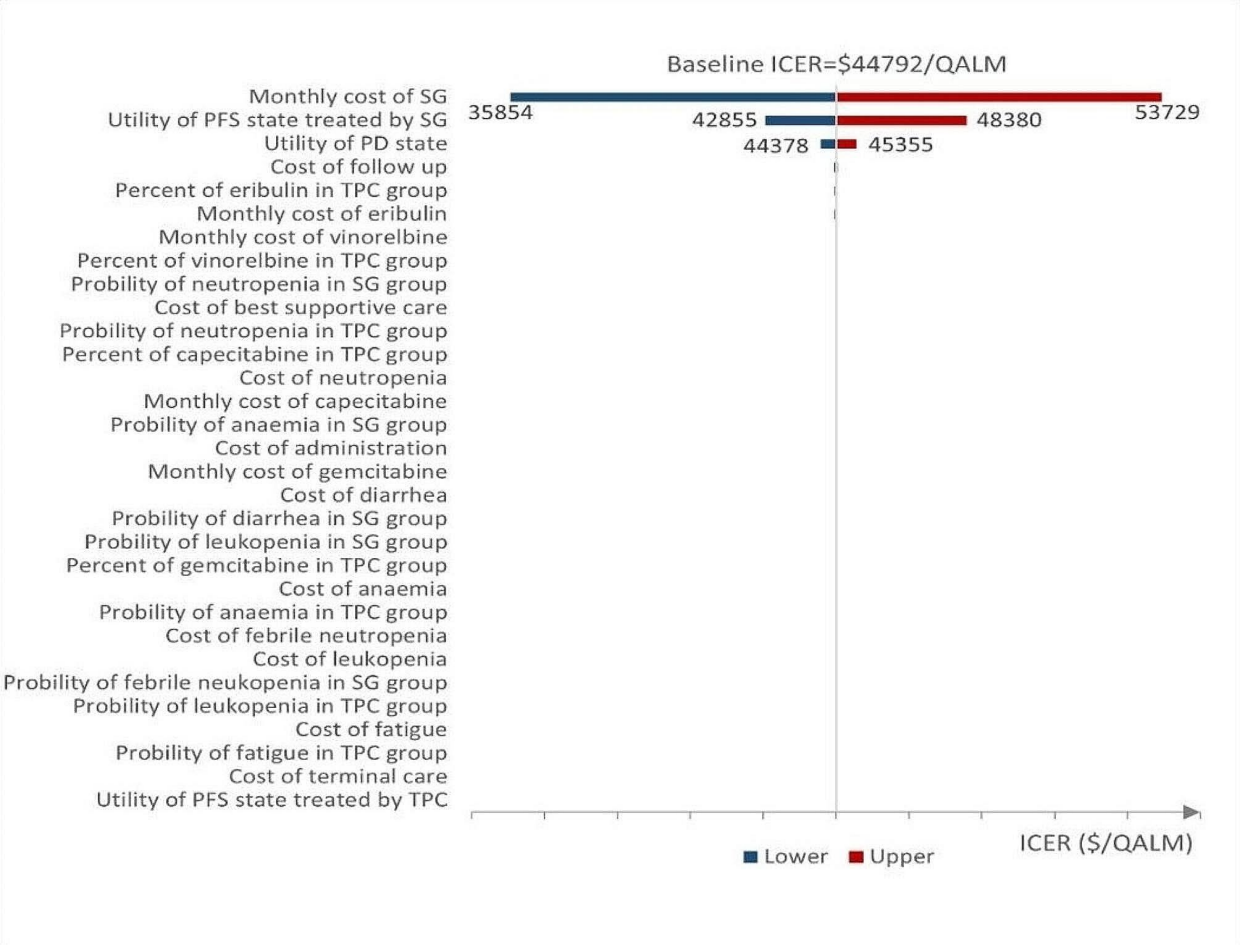
Tornado diagram of one-way sensitive analysis. (Note: SG, Sacituzumab govitecan; TPC, single-agent chemotherapy of physician’s choice; ICER, incremental cost-effectiveness ratio)

**Fig. 4:**
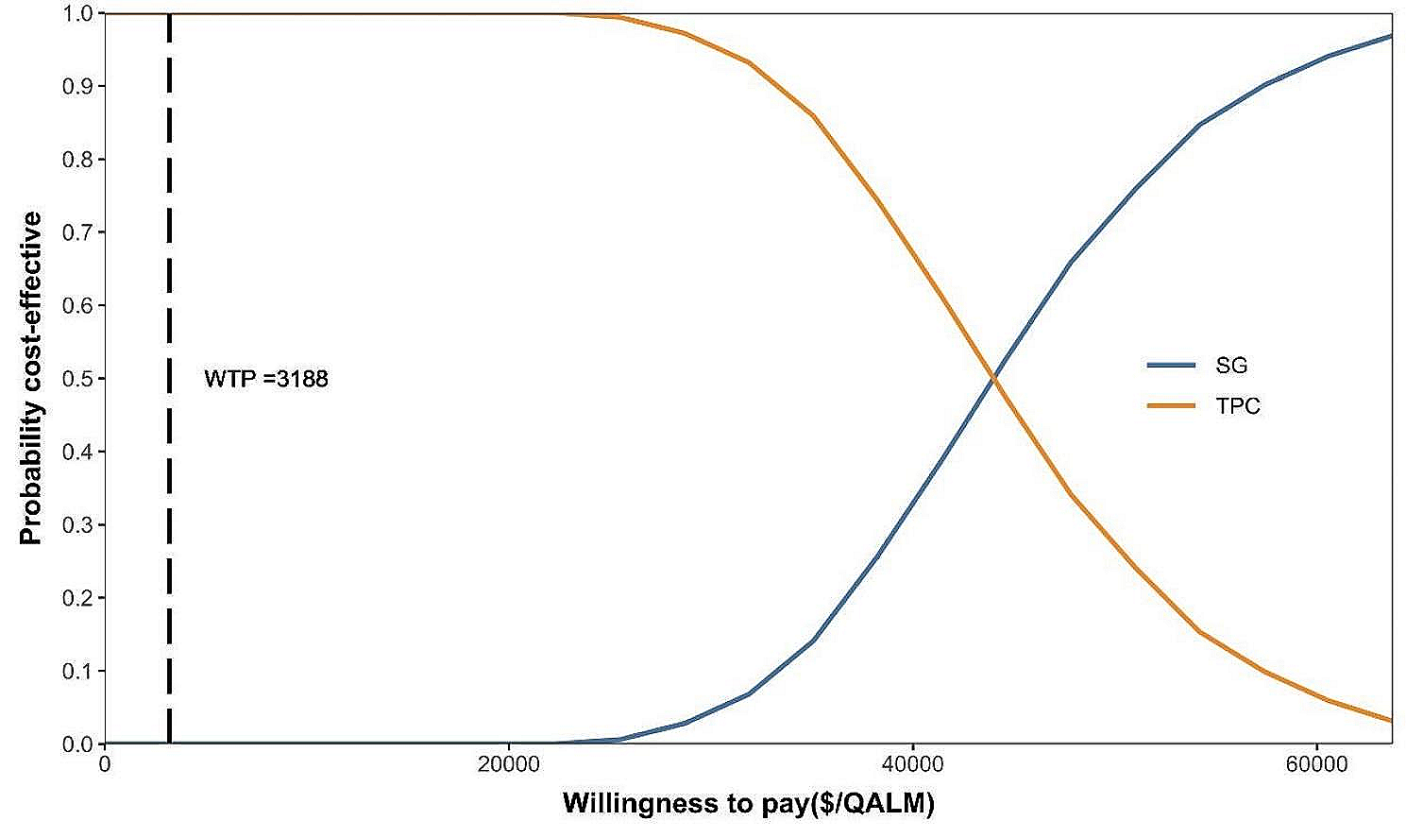
Cost-effective acceptability curves of SG and TPC. (Note: SG, Sacituzumab govitecan; TPC, single-agent chemotherapy of physician’s choice; WTP, willing-to-pay; QALM, quality adjusted life month)

The one-way sensitivity analysis revealed that the cost of SG was a crucial factor for the ICER, followed by the PFS utility value of SG and the PD utility value, as shown in the tornado diagram (Fig. 3). However, the ICER was still much higher than the WTP threshold although these factors varied over a wide range which indicates that the base-case results were sufficiently robust to support the cost-effectiveness conclusion. Other parameters, such as the cost of follow up, AEs, and chemotherapy, had a minor impact on the ICER. The cost-effective acceptability curves of the probabilistic sensitivity analysis showed that the probability of SG being cost-effective was 0% compared with TPC at the WTP threshold of $3188 per QALM in China (Fig. 4).

### Scenario analysis

First, HRs for PFS differed among the subgroups reported in ASCENT clinical trial, we examined the cost-effectiveness of SG regimen in different subgroups. The results suggested that SG was not cost-effective for all subpopulations at the current Hong Kong price (Supplementary Tab.S5). Second, considering that the majority of mTNBC patients won’t survive 10 years that assumed in the baseline analysis, we estimated the cost-effectiveness of SG under 1 year, 3 years, 5 years, and 7 years survival in the scenario analysis. The results showed that the ICERs were all higher than willing-to-pay threshold of $3188/QALM in China which indicating that SG was not cost-effective under 1 year, 3 years, 5 years, and 7 years survival (Table 3). Thirdly, by varying the cost of SG, a threshold price of SG was explored. The results showed that the value of ICER approximately equals WTP ($3188/QALM) when the monthly cost of SG reduced to $2298, the SG regimen become equally cost-effective compare with TPC, the cost-effectiveness acceptability of SG was about 50% in 1000 Monte Carlo simulations (supplementary Fig.S4, Fig.S5). That means SG will be a dominant treatment option for mTNBC patients in Chinese mainland at this price.

**Table 3:** Results of scenario analyses at 1 years, 3 years, 5 years, 7 years of time horizon

Time horizon	TPC		SG		Incremental		ICER*($/QALM)
	QALM	Cost	QALM	Cost	QALM	Cost	
1 year	5.66	4950	7.74	160,212	2.08	155,262	74,487
3 years	7.09	6407	11.77	224,631	4.68	218,224	46,648
5 years	7.12	6441	12.24	236,064	5.12	229,623	44,849
7 years	7.12	6442	12.29	237,683	5.16	231,241	44,795

^*^Compared to TPC at a willing-to-pay of $3188/QALM in China.

Note: SG, Sacituzumab govitecan; TPC, single-agent treatment of physician’s choice; ICER, incremental cost-effectiveness ratio; QALM, quality adjusted life month.

## Discussion

In recent years, the pace of approval and marketing of the latest global antitumor drugs in Chinese mainland has accelerated. However, the introduction of new anticancer drugs is often accompanied by considerable cost increases. The issue of financial toxicity resulting from patented drugs is a pertinent topic, particularly in China, the world’s largest developing country. Despite several rounds of negotiations concerning patented antitumor drugs, China still faces a considerable burden in addressing the growing financial toxicity. As the initial ADC medication approved for post-line treatment of mTNBC, SG exhibited a noteworthy enhancement in terms of PFS and OS in comparison to conventional chemotherapy. However, in the ASCENT trial, the AEs of SG were more frequent than those of chemotherapy, particularly myelosuppression and diarrhea. In essence, SG significantly ameliorates therapeutic outcomes while augmenting the expenses associated with managing AEs. The advantages and disadvantages merit careful consideration. Furthermore, the issue of cost-effectiveness for patients residing in underdeveloped regions to procure medications at prices equivalent to those in developed regions is a major concern due to accessibility. In practice, it is imperative to establish a cost-effective pricing strategy in Chinese mainland to serve as a benchmark for future negotiations. In this study, we estimated the cost-effectiveness of SG compared with single-agent chemotherapy based on its price in Hong Kong, and concluded that SG is not a cost-effective treatment option for patients with mTNBC from a Chinese healthcare system perspective, unless a significant price reduction or preferential drug policy is formulated.

To our knowledge, this is the first pharmacoeconomic study of SG that taking the QoL benefit of the SG regimen into account for mTNBC patients in a Chinese mainland setting. Prior to this, only one similar evaluation in a Chinese setting was carried out by Chen [26]. However, it should be noted that the adopted utility values were based on the United States population and did not consider the QoL benefit in the SG group, which may have underestimated the treatment benefit of SG as a novel therapy compared to chemotherapy. In CEA of tumor-related interventions, an underestimated quality-adjusted life utility will inevitably lead to an overestimated of ICER, which ultimately may result in a negative resource allocation decision. As recently reported [12], SG was superior to chemotherapy on global health status and QoL, physical functioning, fatigue, and pain. Generally, SG was associated with greater benefits in health-related QoL than chemotherapy, which indicates that it is not appropriate to use undistinguished utility values in a health economic evaluation. Therefore, a reanalysis is strongly recommended using the most recently reported QoL data. In our study, firstly, a robust mapping method using adjusted, limited dependent variable mixture model [13] was utilized to convert the scores of the EORTC QLQ-C30 questionnaire collected in the ASCENT trials into preference-based EQ-5D-3 L values that could be used directly in the cost-effectiveness evaluation. Secondly, the incremental utility was added to baseline utility value in Chinese women with breast cancer [11] to form the utility value of the SG regimen in Chinese patients. Finally, different PFS utility value parameters that indicating distinguished benefits of SG and TPC were respectively brought into the PSM model to calculate. Ultimately, the base-case results showed that the ICER ($44,792/QALM) is much higher than the WTP threshold of $3188/QALM in China. It can be concluded that SG was not cost-effective compared with TPC for post-line treatment for mTNBC in China by the current price in HK, despite superior performance in improving QoL. In order to verify the robustness of the conclusion, we performed deterministic sensitivity analysis, probability sensitivity analysis, and scenario analyses respectively, the results proved that the fluctuation of parameters within given range won’t affect the certainty of the conclusion.

Our conclusion is consistent with those of previously published studies. Lang [27] argued that SG is unlikely to be a preferred option at the price of $30.354/2.5 mg for patients with mTNBC compared with TPC from a United States payer perspective. Similarly, Chen [26] evaluated the cost-effectiveness of SG versus TPC from the perspective of the Chinese healthcare system and the United States payer. The results suggested that SG is not cost-effective at a price of RMB192.5/mg in China or $11.2/mg in the US. Xie and colleagues [28] developed a microsimulation model to estimate the cost-effectiveness of SG from US payer perspective, they also found that the price of SG was the most influential factor for the model outcomes and SG was cost-effective only if 80% price reduction at US WTP threshold of $150,000/QALYs compared with chemotherapy. A similar conclusion was also reached in the United Kingdom. According to NICE, the price of SG is £793.00 per 180 mg vial in the UK, which is approximately $5.46/mg. NICE states that, SG is recommended only when the company provides the drug according to the commercial arrangement, but the size of the discount is commercially confidential [29]. In fact, we also conducted a preliminary investigation of the cost-effective price in the Chinese mainland context, which concluded that the SG regimen will be a preferred strategy if its price is reduced to $1.32/mg, which is almost 25% of the current UK price. In a way, NICE’s conclusion confirms our calculation of the cost-effective price. Cher and colleagues [30] assess the cost-effectiveness of SG for mTNBC in Singapore from a healthcare system perspective over 5 years. They also concluded that the ICER was most sensitive to cost of SG and PFS utility values and a substantial price reduction was required to reduce the ICER.

Inevitably, there are several limitations to this analysis. First, this work was based on the results reported in the ASCENT trial to evaluate the cost-effectiveness in the Chinese context. Individual patient data were inaccessible and had to be reconstructed according to Guyot’s algorithm, which might result in uncertainty. However, the method has been confirmed to have significantly less bias and better precision than others [31, 32] and has been widely recommended for economic evaluation [33, 34]. Second, optimal models were used to estimate the survival profile beyond the follow-up period. As with all RCT-based cost-effectiveness analyses, there was model extrapolation bias, introducing a degree of uncertainty into the results. The analysis needs to be further confirmed by real-world data. Third, 18 Asian population cohorts, 3.8% of the total eligible participants, were enrolled in the ASCENT trial, which means that related results may be unrepresentative in China. Fortunately, EVER-132-001 (NCT04454437), a multicenter, single-arm, phase IIb study was recently conducted to validate the efficacy and safety of SG in heavily pretreated Chinese patients with mTNBC. According to the latest report [35], SG provides a comparable benefit and safety profile for Chinese patients as previously reported in ASCENT and no unexpected safety issues were observed. Finally, SG is almost exclusively for patients with mTNBC after multiple-line treatment, and the post-progression regimen is thus rather limited and not mentioned in ASCENT. In our analysis, only follow-up and BSC costs were included in the post-progression costs. Notably, four single-agent chemotherapy regimens were selected as the control group in the ASCENT trial, which also confirmed that there are almost no standard post-line treatments for mTNBC from a clinical practice perspective. Although a small percentage of patients may have received post-line therapies not recommended by guidelines, their real-world proportion is difficult to determine. It is expected that more high-quality real-world data will be produced to support pharmacoeconomic research and future decision-making. We also suggested the necessity for a cost-effectiveness reanalysis of SG if potential post-line therapies emerge in the future.

## Conclusion

SG was estimated unlikely to be a cost-effective option for patients with mTNBC in China at a WTP threshold of $3188 per QALM from the perspective of the Chinese health system.

### Electronic supplementary material

Below is the link to the electronic supplementary material.

Supplementary Material 1

## Data Availability

All data generated or analysed during this study are included in this published article and its supplementary information files.

## Abbreviations

ADC: Antibody-drug conjugate
AEs: Adverse events
AIC: Akaike’s Information Criteria
BIC: Bayesian Information Criterion
BSC: Best supportive care
CHEERS: The Consolidated Health Economic Evaluation Reporting Standards
CPI: Consumer Price Index
CSCO: Chinese Society of Clinical Oncology
GDP: Gross domestic product
HER-2: Human epidermal growth factor receptor-2
HR: Hazard ratio
ICER: Incremental cost-effectiveness ratios
INMB: Incremental net monetary benefit
IPD: Individual patient data
mTNBC: Metastatic triple-negative breast cancer
NMB: Net monetary benefit
OS: Overall survival
PD: Progressive disease
PFS: Progression-free survival
PSA: Probabilistic sensitivity analyses
PSM: Partitioned survival model
QoL: Quality of life
QALM: Quality-adjusted life month
SG: Sacituzumab govitecan
TPC: Treatment of physician’s choice
Trop-2: Trophoblast cell-surface antigen 2
WTP: Willingness-to-pay

## References

[1] Sung H, Ferlay J, Siegel RL, Laversanne M, Soerjomataram I, Jemal A, Bray F (2021). Global Cancer statistics 2020: GLOBOCAN estimates of incidence and Mortality Worldwide for 36 cancers in 185 countries. CA Cancer J Clin.

[2] Garrido-Castro AC, Lin NU, Polyak K (2019). Insights into Molecular classifications of Triple-negative breast Cancer: improving patient selection for treatment. Cancer Discov.

[3] Chai J, Hu J, Wang T, Bao X, Luan J, Wang Y (2024). A multifunctional liposome for synergistic chemotherapy with ferroptosis activation of Triple-negative breast Cancer. Mol Pharm.

[4] Foulkes WD, Smith IE, Reis-Filho JS (2010). Triple-negative breast cancer. N Engl J Med.

[5] Cortes J, Rugo HS, Cescon DW, Im SA, Yusof MM, Gallardo C, Lipatov O, Barrios CH, Perez-Garcia J, Iwata H (2022). Pembrolizumab plus Chemotherapy in Advanced Triple-negative breast Cancer. N Engl J Med.

[6] Goldenberg DM, Cardillo TM, Govindan SV, Rossi EA, Sharkey RM. Trop-2 is a novel target for solid cancer therapy with sacituzumab govitecan (IMMU-132), an antibody-drug conjugate (ADC)*. Oncotarget 2015, 6. 10.18632/oncotarget.4318 PMC4673178 26101915

[7] Bardia A, Hurvitz SA, Tolaney SM, Loirat D, Punie K, Oliveira M, Brufsky A, Sardesai SD, Kalinsky K, Zelnak AB (2021). Sacituzumab Govitecan in Metastatic Triple-negative breast Cancer. N Engl J Med.

[8] China Guidelines for Pharmacoeconomic Evaluations. (2020) [https://www.ispor.org/heor-resources/more-heor-resources/pharmacoeconomic-guidelines/pe-guideline-detail/china-mainland] Accessed 20 May 2013.

[9] Guyot P, Ades AE, Ouwens MJ, Welton NJ (2012). Enhanced secondary analysis of survival data: reconstructing the data from published Kaplan-Meier survival curves. BMC Med Res Methodol.

[10] Woods BS, Sideris E, Palmer S, Latimer N, Soares M (2020). Partitioned survival and State Transition Models for Healthcare decision making in Oncology: where are we now?. Value Health.

[11] Rautenberg T, Hodgkinson B, Zerwes U, Downes M (2022). Meta-analysis of health state utility values measured by EuroQol 5-dimensions (eq. 5D) questionnaire in Chinese women with breast cancer. BMC Cancer.

[12] Loibl S, Loirat D, Tolaney SM, Punie K, Oliveira M, Rugo HS, Bardia A, Hurvitz SA, Brufsky AM, Kalinsky K (2023). Health-related quality of life in the phase III ASCENT trial of sacituzumab govitecan versus standard chemotherapy in metastatic triple-negative breast cancer. Eur J Cancer.

[13] Gray LA, Hernandez Alava M, Wailoo AJ (2021). Mapping the EORTC QLQ-C30 to EQ-5D-3L in patients with breast cancer. BMC Cancer.

[14] The National Physical Fitness Monitoring Center issued the Fifth National Physical. Fitness Monitoring Communique [https://www.sport.gov.cn/n315/n329/c24335066/content.html] Accessed 20 May 2013.

[15] National Bureau of Statistics of China. [https://data.stats.gov.cn/easyquery.htm?cn=C01] Accessed 20 May 2013.

[16] People’s Bank of China. [http://www.pbc.gov.cn/rmyh/108976/109428/index.html] Accessed 20 May 2013.

[17] DrugsHK. [https://drugs-hk.squarespace.com/products-en] Accessed 20 May 2013.

[18] Yaozhi database. [https://db.yaozh.com/yaopinzhongbiao?comprehensivesearchcontent] Accessed 20 May 2013.

[19] Committee CSoCOGW (2017). [Guidelines for standardized management of neutropenia induced by chemotherapy and radiotherapy]. Zhonghua Zhong Liu Za Zhi.

[20] Dranitsaris G, Yu B, King J, Kaura S, Zhang A (2015). Nab-paclitaxel, docetaxel, or solvent-based paclitaxel in metastatic breast cancer: a cost-utility analysis from a Chinese health care perspective. Clinicoecon Outcomes Res.

[21] Li J, Jiang Z (2022). Chinese Society of Clinical Oncology Breast Cancer (CSCO BC) guidelines in 2022: stratification and classification. Cancer Biol Med.

[22] Wu B, Zhang Q, Sun J (2018). Cost-effectiveness of nivolumab plus ipilimumab as first-line therapy in advanced renal-cell carcinoma. J Immunother Cancer.

[23] Patel KK, Isufi I, Kothari S, Davidoff AJ, Gross CP, Huntington SF (2020). Cost-effectiveness of first-line vs third-line ibrutinib in patients with untreated chronic lymphocytic leukemia. Blood.

[24] Wan X, Zhang Y, Tan C, Zeng X, Peng L (2019). First-line Nivolumab Plus Ipilimumab vs Sunitinib for metastatic renal cell carcinoma: a cost-effectiveness analysis. JAMA Oncol.

[25] Husereau D, Drummond M, Augustovski F, de Bekker-Grob E, Briggs AH, Carswell C, Caulley L, Chaiyakunapruk N, Greenberg D, Loder E (2022). Consolidated Health Economic Evaluation Reporting Standards (CHEERS) 2022 explanation and elaboration: a report of the ISPOR CHEERS II Good practices Task Force. Value Health.

[26] Chen J, Han M, Liu A, Shi B (2021). Economic evaluation of Sacituzumab Govitecan for the treatment of metastatic triple-negative breast Cancer in China and the US. Front Oncol.

[27] Lang Y, Chai Q, Tao W, Liao Y, Liu X, Wu B (2023). Cost-effectiveness of sacituzumab govitecan versus chemotherapy in advanced or metastatic triple-negative breast cancer. Breast.

[28] Xie J, Li S, Li Y, Li J (2023). Cost-effectiveness of sacituzumab govitecan versus chemotherapy in patients with relapsed or refractory metastatic triple-negative breast cancer. BMC Health Serv Res.

[29] Sacituzumab govitecan for. treating unresectable triple-negative advanced breast cancer after 2 or more therapies [https://www.nice.org.uk/guidance/ta819/chapter/2-Information-about-sacituzumab-govitecan] Accessed 20 May 2013. 40504964

[30] Cher BP, Goh S, Aziz MIA, Wong G, Ng Chee Hui R, Ong BS, Ng KH (2024). Cost-utility analysis of sacituzumab govitecan versus chemotherapy for the treatment of metastatic triple-negative breast cancer in Singapore. Expert Rev Pharmacoecon Outcomes Res.

[31] Wan X, Peng L, Li Y (2015). A review and comparison of methods for recreating individual patient data from published Kaplan-Meier survival curves for economic evaluations: a simulation study. PLoS ONE.

[32] Saluja R, Cheng S, Delos Santos KA, Chan KKW (2019). Estimating hazard ratios from published Kaplan-Meier survival curves: a methods validation study. Res Synth Methods.

[33] Cao X, Zhang M, Li N, Zheng B, Liu M, Song X, Cai H (2023). First-line nivolumab plus chemotherapy versus chemotherapy alone for advanced gastric cancer, gastroesophageal junction cancer, and esophageal adenocarcinoma: a cost-effectiveness analysis. Ther Adv Med Oncol.

[34] Yang J, Han J, Zeng N, Yan X (2023). Cost-effectiveness of trastuzumab deruxtecan in previously treated human epidermal growth factor receptor 2-low metastatic breast cancer. Ther Adv Med Oncol.

[35] Xu B, Ma F, Wang T, Wang S, Tong Z, Li W, Wu X, Wang X, Sun T, Pan Y (2023). A phase IIb, single arm, multicenter trial of sacituzumab govitecan in Chinese patients with metastatic triple-negative breast cancer who received at least two prior treatments. Int J Cancer.

