# Supplementary material for: Cost-effectiveness of sacituzumab govitecan versus single-agent chemotherapy for metastatic triple-negative breast cancer: a trial-based analysis
Source: Cost Eff Resour Alloc. 2024 Apr 24;22:32. doi: 10.1186/s12962-024-00539-y (PMC11044338; doi:10.1186/s12962-024-00539-y)
Supplement: Supplementary file 1 — Supplementary Material 1 [file 12962_2024_539_MOESM1_ESM.docx]

## Supplementary Figures

**Fig.S1** Reconstructed Kaplan-Meier OS Curves in ACENT trial

**Fig.S2** Reconstructed Kaplan-Meier PFS Curves in ACENT trial

**Fig.S3** The model fitting results of Kaplan-Meier curves of PFS and OS in ACENT trial

**Fig.S4** Incremental cost-effectiveness scatterplot of SG and TPC (SG monthly cost = $2298)

**Fig.S5** Cost-effectiveness acceptability curve of SG and TPC (SG monthly cost = $2298)

**Fig.S6** Plot of probability distribution of INMB under 100/1000/10000 Monte Carlo simulations

## Supplementary Tables

**Tab.S1** Summary of estimated parameters and AIC/BIC values of PFS and OS models

**Tab.S2** The related parameters in EQ-5D utility value calculation

**Tab.S3** The doses and costs of drugs

**Tab.S4** Probability of adverse events in ACENT trial (Grade≥3, rate≥5)

**Tab.S5** Subgroup analysis of ICER and probabilities of cost-effectiveness

**Tab.S6** CHEERS 2022 Checklist

**Tab.S7** Summary of annual survival probabilities in the partitional survival model

## Supplementary Figures


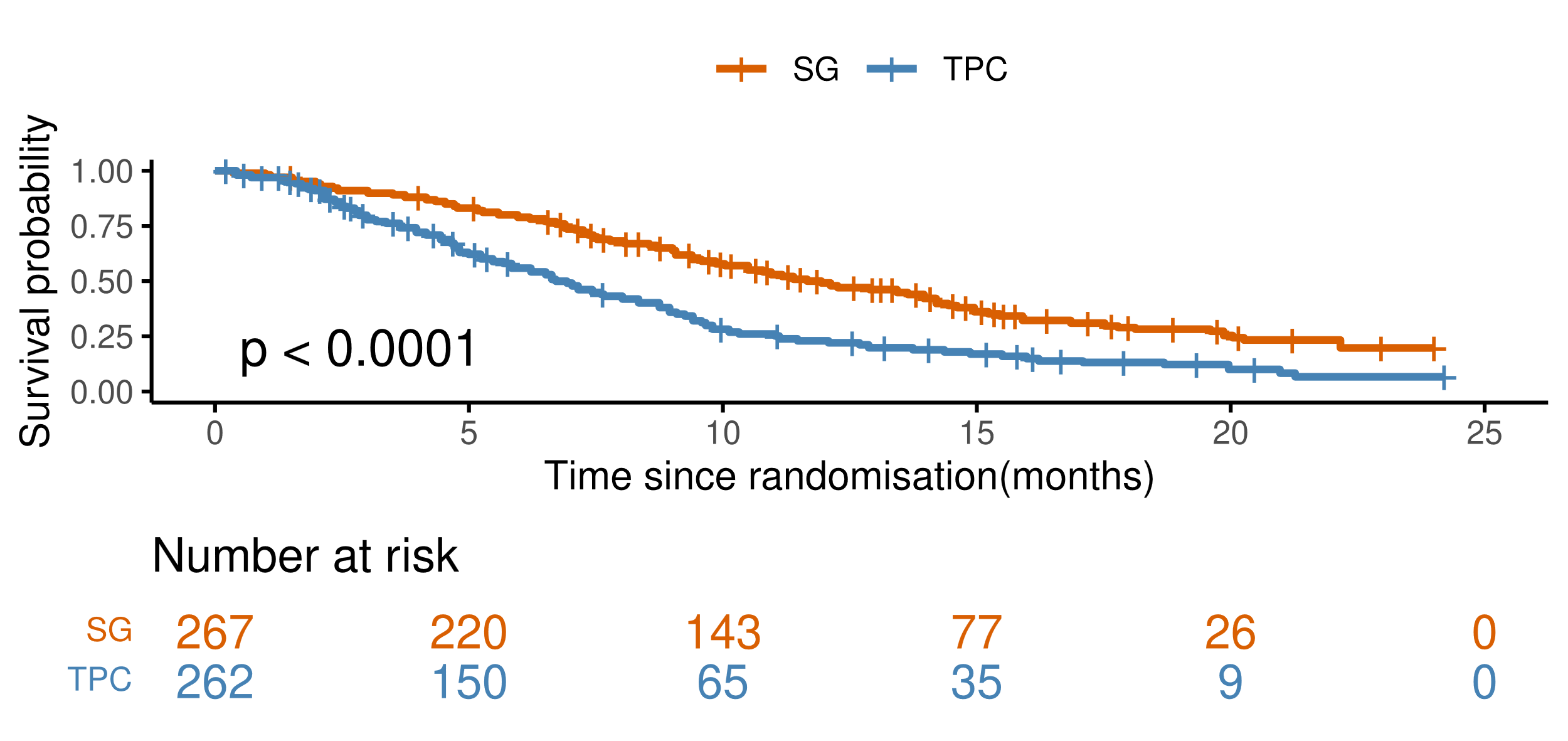


**Fig.S1** Reconstructed Kaplan-Meier OS Curves in ACENT trial

Note: SG, sacituzumab govitecan; TPC, single-agent chemotherapy of physician’s choice; OS, overall survival.


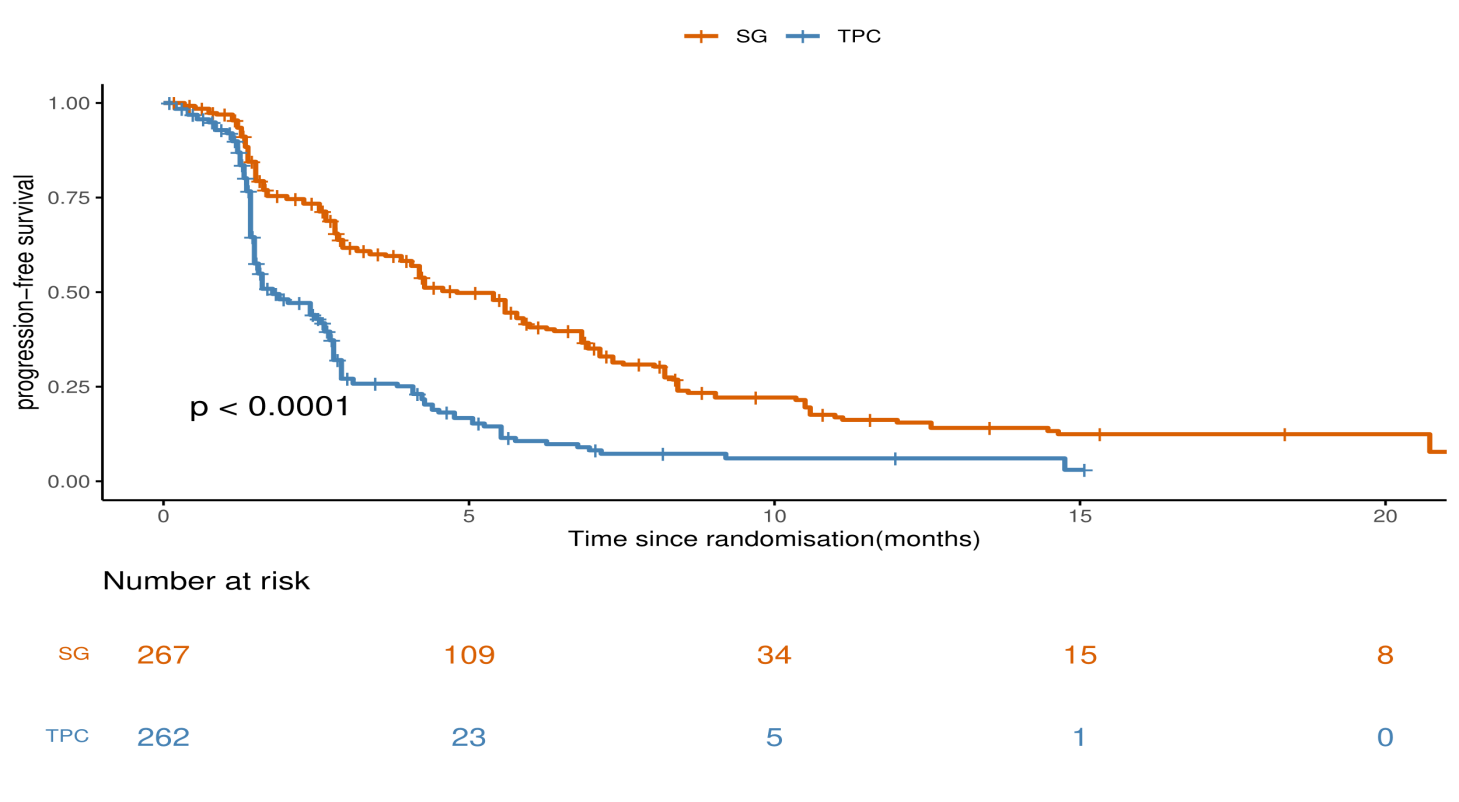


**Fig.S2** Reconstructed Kaplan-Meier PFS Curves in ACENT trial

Note: SG, sacituzumab govitecan; TPC, single-agent chemotherapy of physician’s choice; PFS, progression-free survival.


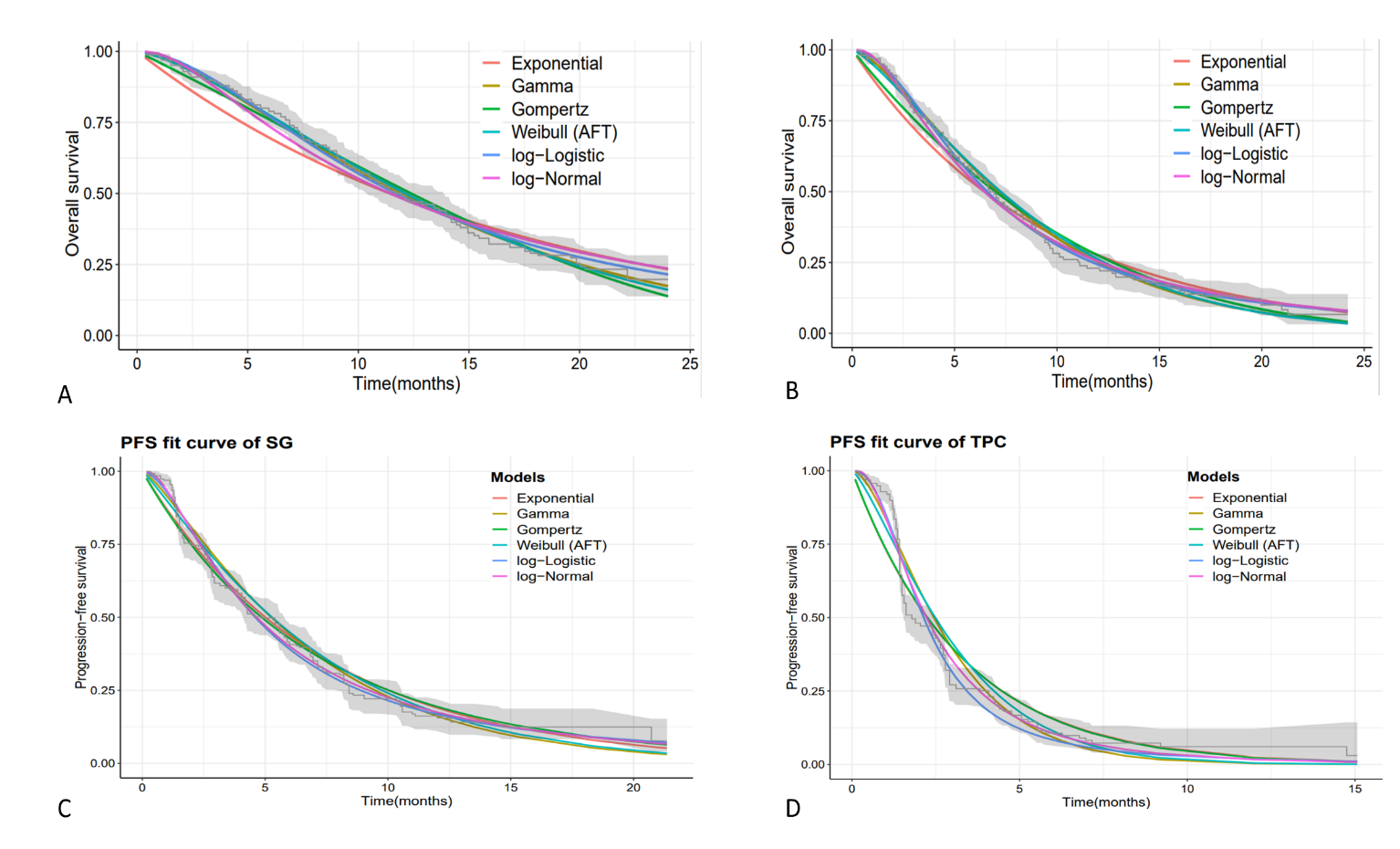


**Fig.S3** The model fitting results of Kaplan-Meier curves of PFS and OS in ACENT trial.

Figure A, B represent the model fitting of the OS curves of SG and TPC, respectively. Figure C, D show the model fitting of the PFS curves of SG and TPC, respectively. The gray area represents the 95% confidence interval for each Kaplan-Meier curve.

Note: SG, sacituzumab govitecan; TPC, single-agent chemotherapy of physician’s choice; PFS, progression-free survival; OS, overall survival.


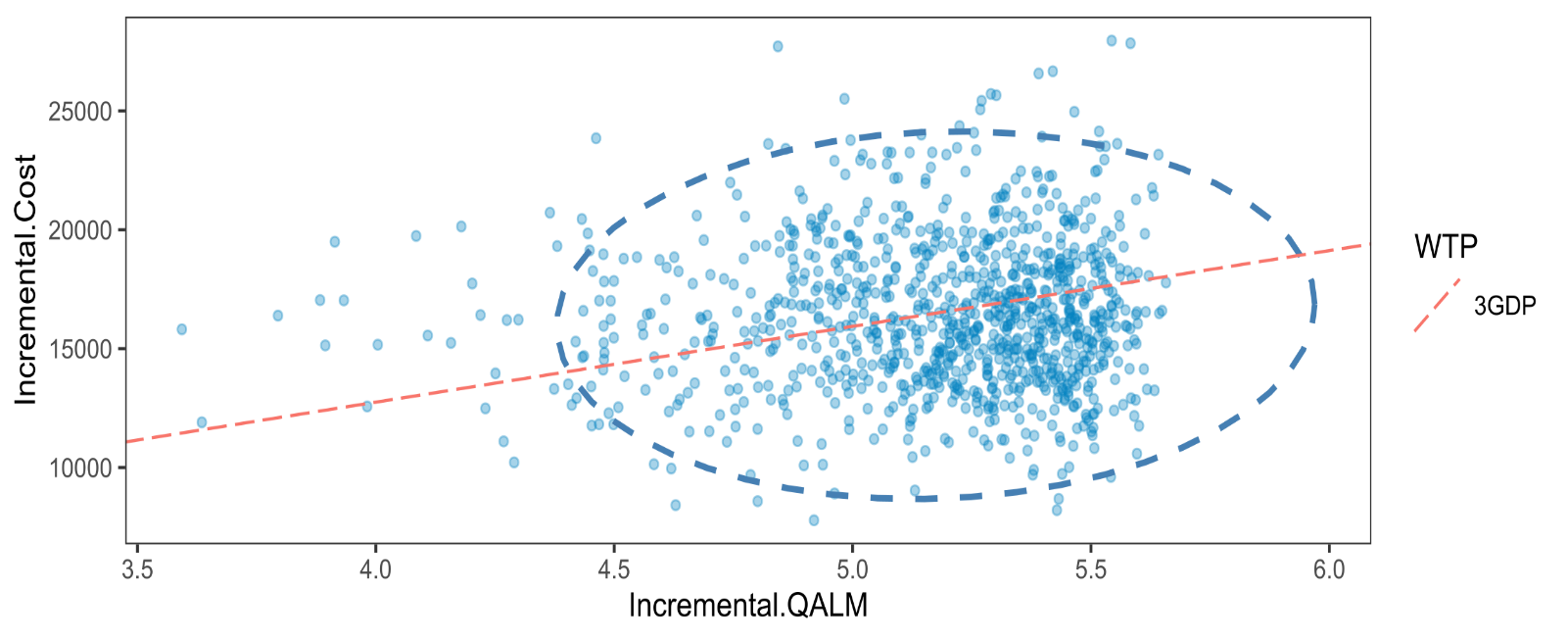


**Fig.S4** Incremental cost-effectiveness scatterplot of SG and TPC (SG monthly cost = $2298)

Note:WTP, willing-to-pay; QALM, quality adjusted life month. The blue dashed ellipse represents the 95% confidence interval.


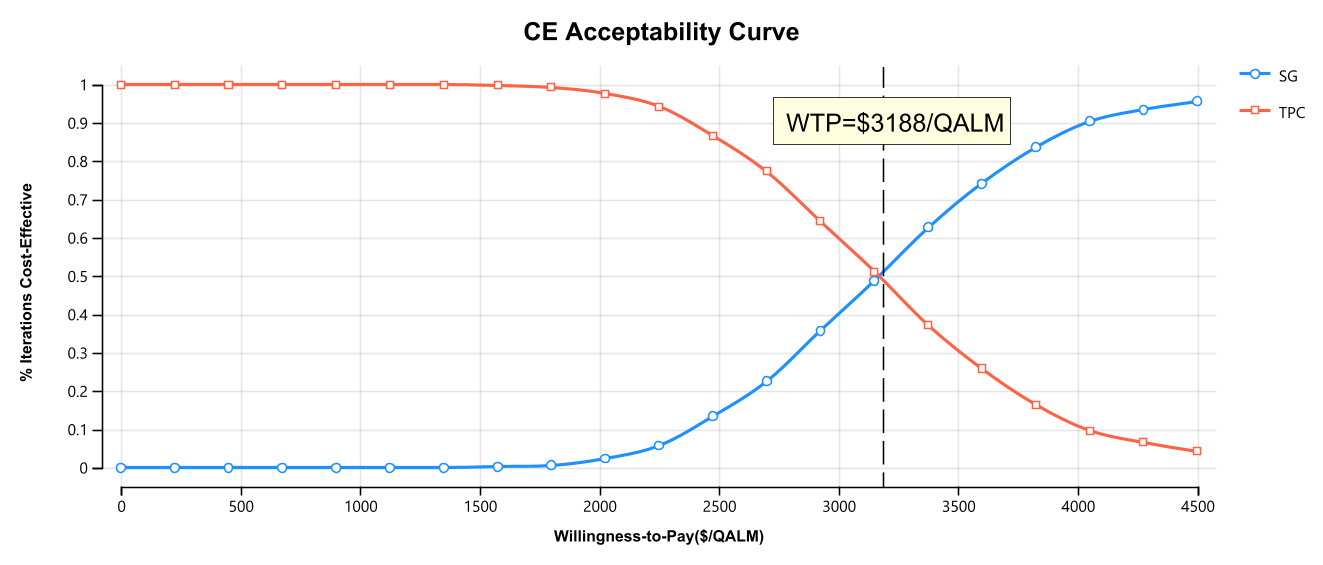


**Fig.S5** Cost-effectiveness acceptability curve of SG and TPC (SG monthly cost = $2298)

Note:WTP, willing-to-pay; QALM, quality adjusted life month.


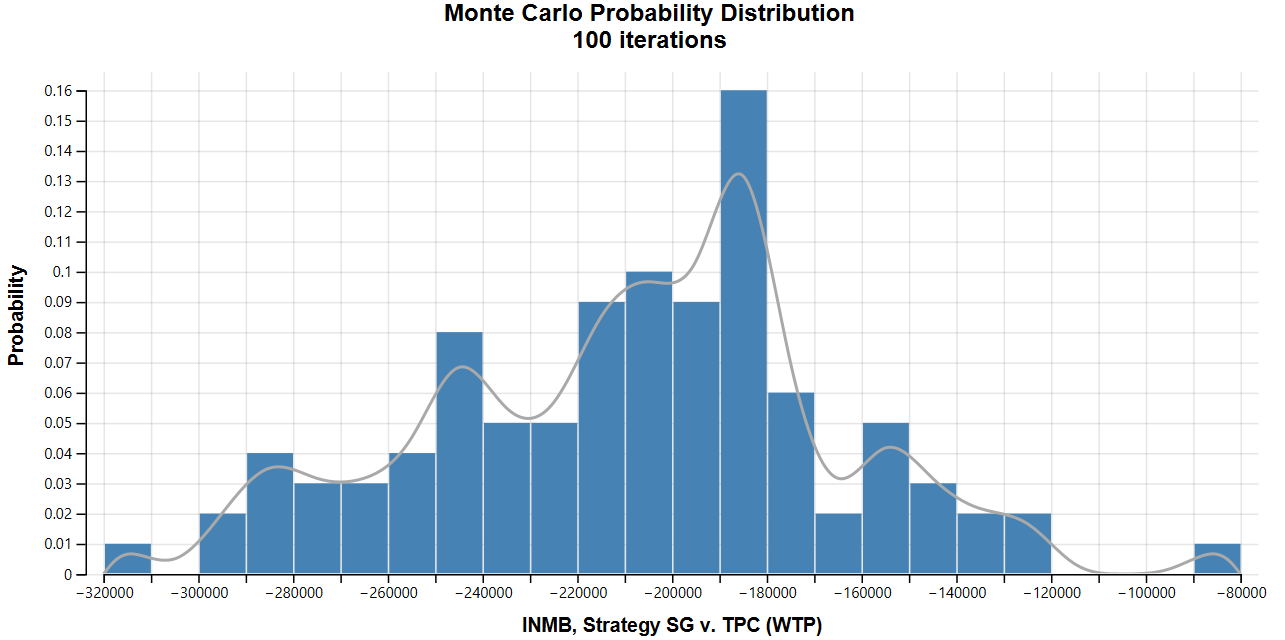


B.

A.


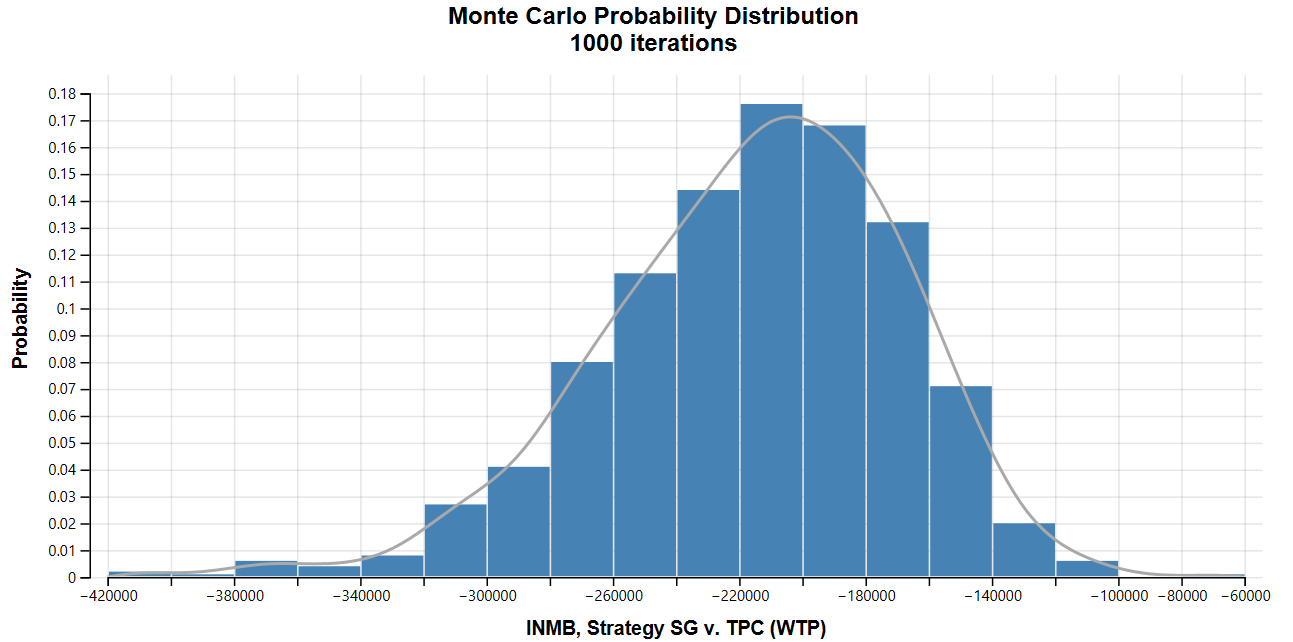


C.


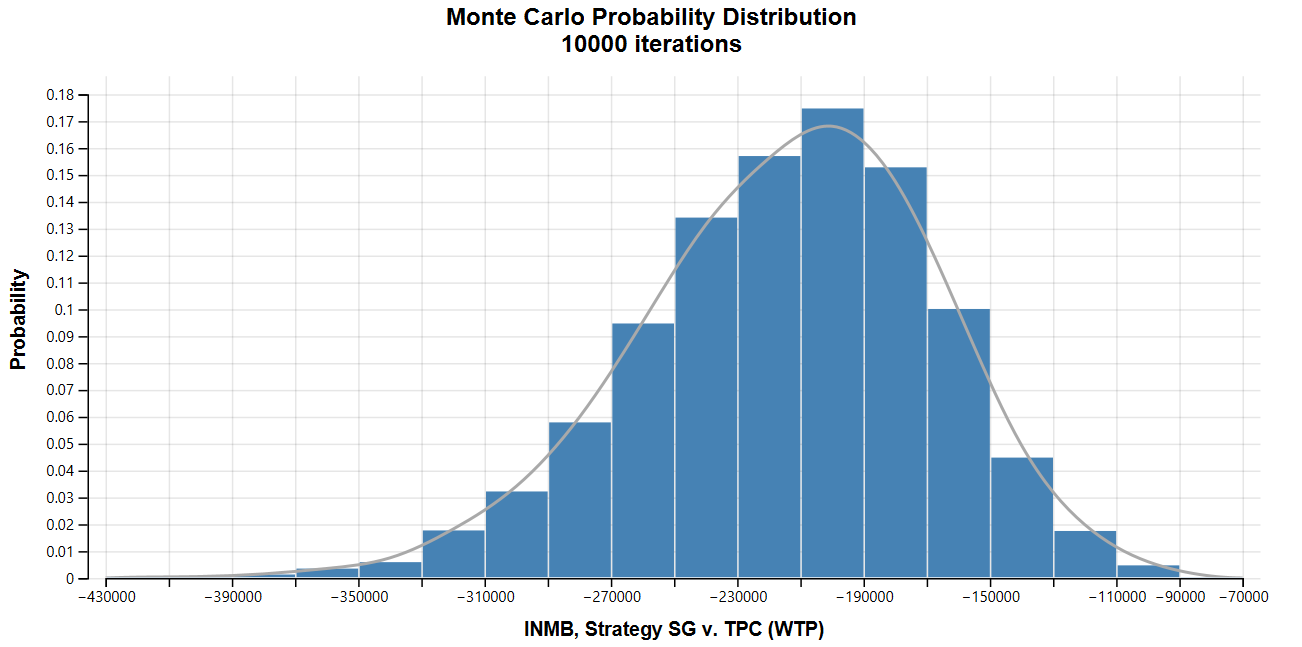


**Fig.S6** Plot of probability distribution of INMB under 100/1000/10000 Monte Carlo simulations

Note: SG, sacituzumab govitecan; TPC, single-agent chemotherapy of physician’s choice; WTP, willing-to-pay; INMB, incremental net monetary benefit.

A:100 simulations, B: 1000 simulations, C: 10000 simulations.

## Supplementary Tables

**Tab.S1** Summary of estimated parameters and AIC/BIC values of PFS and OS models.

| Strategies | Distributions | Parameters | PFS | | | | | | OS | | | | | |
| --- | --- | --- | --- | --- | --- | --- | --- | --- | --- | --- | --- | --- | --- | --- |
|  |  |  | Est | L95% | U95% | SE | AIC | BIC | Est | L95% | U95% | SE | AIC | BIC |
| SG | Exponential | rate | 0.13800 | 0.11984 | 0.15891 | 0.00993 | 1152.46 | 1156.05 | 0.06045 | 0.05226 | 0.06993 | 0.00449 | 1379.74 | 1383.32 |
|  | Gamma | shape | 1.3247 | 1.1155 | 1.5733 | 0.1162 | 1144.87 | 1152.05 | 1.6587 | 1.3787 | 1.9955 | 0.1565 | 1356.14 | 1363.32 |
|  |  | rate | 0.1920 | 0.1522 | 0.2422 | 0.0228 |  |  | 0.1125 | 0.0880 | 0.1437 | 0.0141 |  |  |
|  | Gompertz | shape | -0.0125 | -0.0451 | 0.0201 | 0.0166 | 1153.88 | 1161.06 | 0.05727 | 0.03162 | 0.08293 | 0.01309 | 1363.48 | 1370.65 |
|  |  | rate | 0.1462 | 0.1192 | 0.1793 | 0.0152 |  |  | 0.03807 | 0.02901 | 0.04998 | 0.00528 |  |  |
|  | Weibull | shape | 1.1274 | 1.0121 | 1.2559 | 0.0621 | 1150.01 | 1157.18 | 1.4178 | 1.2510 | 1.6069 | 0.0905 | 1355.91* | 1363.08* |
|  |  | scale | 7.2931 | 6.4330 | 8.2682 | 0.4669 |  |  | 15.6718 | 14.1329 | 17.3781 | 0.8264 |  |  |
|  | Log-logistic | shape | 1.6659 | 1.4858 | 1.8678 | 0.0972 | 1125.29 | 1132.47 | 1.801 | 1.590 | 2.041 | 0.115 | 1359.64 | 1366.81 |
|  |  | scale | 4.5927 | 4.0198 | 5.2473 | 0.3122 |  |  | 11.680 | 10.374 | 13.149 | 0.706 |  |  |
|  | Log-normal | meanlog | 1.5362 | 1.4051 | 1.6673 | 0.0669 | 1118.25* | 1125.43* | 2.4372 | 2.3037 | 2.5707 | 0.0681 | 1371.88 | 1379.05 |
|  |  | sdlog | 1.0136 | 0.9152 | 1.1226 | 0.0528 |  |  | 1.0251 | 0.9197 | 1.1427 | 0.0568 |  |  |
| TPC | Exponential | rate | 0.3087 | 0.2675 | 0.3563 | 0.0226 | 815.58 | 819.15 | 0.10709 | 0.09357 | 0.12256 | 0.00737 | 1366.79 | 1370.36 |
|  | Gamma | shape | 1.8637 | 1.5601 | 2.2266 | 0.1691 | 777.15 | 784.29 | 1.6149 | 1.3605 | 1.9169 | 0.1412 | 1342.10 | 1349.24 |
|  |  | rate | 0.6306 | 0.5047 | 0.7880 | 0.0717 |  |  | 0.1823 | 0.1472 | 0.2258 | 0.0199 |  |  |
|  | Gompertz | shape | 0.00656 | -0.05007 | 0.06320 | 0.02889 | 817.53 | 824.67 | 0.03155 | 0.00588 | 0.05722 | 0.01310 | 1363.28 | 1370.42 |
|  |  | rate | 0.30433 | 0.25172 | 0.36794 | 0.02947 |  |  | 0.08802 | 0.07084 | 0.10937 | 0.00975 |  |  |
|  | Weibull | shape | 1.2981 | 1.1756 | 1.4335 | 0.0657 | 795.22 | 802.36 | 1.3057 | 1.1734 | 1.4528 | 0.0711 | 1347.69 | 1354.83 |
|  |  | scale | 3.2858 | 2.9396 | 3.6727 | 0.1866 |  |  | 9.5755 | 8.6262 | 10.6293 | 0.5101 |  |  |
|  | Log-logistic | shape | 2.325 | 2.064 | 2.619 | 0.141 | 734.60* | 741.74* | 1.895 | 1.695 | 2.119 | 0.108 | 1335.71* | 1342.85* |
|  |  | scale | 2.135 | 1.937 | 2.353 | 0.106 |  |  | 6.578 | 5.869 | 7.373 | 0.383 |  |  |
|  | Log-normal | meanlog | 0.7994 | 0.6946 | 0.9043 | 0.0535 | 746.37 | 753.51 | 1.8650 | 1.7464 | 1.9836 | 0.0605 | 1341.01 | 1348.15 |
|  |  | sdlog | 0.7923 | 0.7153 | 0.8776 | 0.0413 |  |  | 0.9356 | 0.8489 | 1.0313 | 0.0465 |  |  |

Note: AIC, Akaike's information criterion; BIC, Bayesian information criterion; SG, sacituzumab govitecan; TPC, single-agent chemotherapy of physician’s choice; Est, estimated value; L95%, lower value in 95% confidence interval; U95%, upper value in 95% confidence interval; SE, standard error.

*The model was used to fit the Kaplan-Meier curve.

**Tab.S2** The related parameters in EQ-5D utility value calculation.

|  | | EORTC QLQ-C30 subscores | | | | | | |
| --- | --- | --- | --- | --- | --- | --- | --- | --- |
| HRQoL domains | | SG | | | TPC | | | |
|  |  | Baseline | LS mean change | Overall score within 6 cycles | Baseline | LS mean change | | Overall score within 6 cycles |
| Global health status/QoL | | 63.2 | 0.66 | 63.86 | 58.1 | -3.42 | 54.68 | |
| Physical functioning | | 74.9 | 1.31 | 76.21 | 73 | -4.39 | 68.61 | |
| Role functioning | | 69.6 | -2.24 | 67.36 | 67.9 | -7.83 | 60.07 | |
| Emotional functioning | | 72.1 | 3.34 | 75.44 | 69.9 | -0.55 | 69.35 | |
| Cognitive functioning | | 82.5 | -1.22 | 81.28 | 80 | -1.98 | 78.02 | |
| Social functioning | | 70.6 | -1.51 | 69.09 | 71.2 | -5.41 | 65.79 | |
| Fatigue | | 38.3 | 1.97 | 40.27 | 40.1 | 7.13 | 47.23 | |
| Nausea/vomiting | | 7.6 | 4.3 | 11.9 | 9.9 | 2.5 | 12.4 | |
| Pain | | 36.4 | -8.93 | 27.47 | 40.3 | -1.89 | 38.41 | |
| Dyspnea | | 24.7 | -3.79 | 20.91 | 25.1 | 3.95 | 29.05 | |
| Insomnia | | 31.6 | -4.69 | 26.91 | 36.1 | 0.34 | 36.44 | |
| Appetite loss | | 19.2 | 3.52 | 22.72 | 24 | 7 | 31 | |
| Constipation | 16.6 | 2.16 | 18.76 | 17.5 | 2.69 | 20.19 | |  |
| Diarrhea | 7.4 | 14.07 | 21.47 | 6.4 | -1.27 | 5.13 | |  |
| Financial difficulties | 27.2 | -2.87 | 24.33 | 23 | 0.68 | 23.68 | |  |
| Age | 54 |  | 54 | 54 |  | 54 | |  |
| Calculated EQ-5D value | 0.679 |  | 0.711 | 0.659 |  | 0.644 | |  |

Note: HRQoL, health-related quality of life; LS, Least-square; SG, Sacituzumab govitecan; TPC, treatment of physician's choice.

**Tab.S3** The doses and costs of drugs

| **Drug** | **Dose** | **Administration regimen** | **Unit price ($)** | **Cost for respective treatment cycle ($)** | **Cost for 1 month ($)** |
| --- | --- | --- | --- | --- | --- |
| SG | 10mg/kg× 60.8kg | Administered intravenously d1, d8 every 3 weeks | 3448.10/180mg | 23293.95 | 33277 |
| Eribulin | 1.4mg/m^2^×1.58m^2^ | Administered intravenously d1, d8 every 3 weeks | 108.02/1mg | 475.24 | 679 |
| Vinorelbine | 25mg/m^2^×1.58m^2^ | Administered intravenously d1 every week | 42.18/10mg | 166.65 | 714 |
| Capecitabine | 1g/m^2^ × 1.58m^2^ | Oral administration of 1g/m2 twice a day for 14 days every 3 weeks | 39.32/6g | 289.85 | 414 |
| Gemcitabine | 1g/m^2^ × 1.58m^2^ | Administered intravenously d1, d8, d15 every 4 weeks | 9.52/0.2g | 225.72 | 242 |

Note: SG, Sacituzumab govitecan

**Tab. S4** Probability of adverse events in ACENT trial (Grade≥3, rate≥5)

| **Risk for main AEs** | Number of patients | Percent(%） |
| --- | --- | --- |
| **SG group** |  |  |
| Neutropenia | 132 | 51.16 |
| Anaemia | 20 | 7.75 |
| Leukopenia | 26 | 10.08 |
| Febrile neutropenia | 15 | 5.81 |
| Diarrhea | 27 | 10.47 |
| **TPC group** | | |
| Neutropenia | 74 | 33.04 |
| Anaemia | 11 | 4.91 |
| Leukopenia | 12 | 5.36 |
| Fatigue | 12 | 5.36 |

Note: SG, Sacituzumab govitecan; TPC, treatment of physician's choice.

**Tab.S5** Subgroup analysis of ICER and probabilities of cost-effectiveness

| **Subgroup** | **Sample size** | **PFS HR (95%CI)** | **ICER (95%CI)** | **Cost-effectiveness probability (%) at WTP=$3188/QALM** |
| --- | --- | --- | --- | --- |
| **Age** | | | | |
| ＜65 yr | 378 | 0.46(0.35-0.59) | 48626(48991-48155) | 0 |
| ≥65 yr | 90 | 0.22(0.12-0.40) | 49359(49580-48830) | 0 |
| **Race** | | | | |
| White | 369 | 0.39(0.30-0.51) | 48863(48449-49141) | 0 |
| Black | 56 | 0.45(0.24-0.86) | 48661(47121-49308) | 0 |
| Asian | 18 | 0.40(0.08-2.08) | 48830(43787-49651) | 0 |
| **Previous therapies** | | | | |
| 2 or 3 | 330 | 0.39(0.29-0.52) | 48863(48413-49171) | 0 |
| ＞3 | 138 | 0.48(0.32-0.72) | 48556(47661-49083) | 0 |
| **Geographic region** | | | | |
| North America | 298 | 0.44(0.33-0.60) | 48695(48118-49052) | 0 |
| Rest of the world | 170 | 0.36(0.24-0.53) | 48959(48376-49308) | 0 |
| **Previous use of PD-1 or PD-L1 inhibitors** | | | | |
| Yes | 127 | 0.37(0.24-0.57) | 48927(48229-49308) | 0 |
| No | 341 | 0.42(0.32-0.56) | 48763(48266-49083) | 0 |
| **Liver metastasis** | | | | |
| Yes | 199 | 0.48(0.34-0.67) | 48556(47853-49022) | 0 |
| No | 269 | 0.36(0.26-0.50) | 48959(48485-49255) | 0 |
| **Initial diagnosis of TNBC** | | | | |
| Yes | 322 | 0.38(0.29-0.51) | 48895(48449-49171) | 0 |
| No | 146 | 0.48(0.32-0.72) | 48556(47661-49083) | 0 |

Note: CI, confidence interval; HR, hazard ratio; ICER, incremental cost-effectiveness ratio; QALM, quality adjusted life month; WTP, willing-to-pay; TNBC, triple negative breast cancer.

# Tab.S6 CHEERS 2022 Checklist

| **Topic** | **Item** | **Guidance for Reporting** | **Reported in section** |
| --- | --- | --- | --- |
| **TITLE** | | | |
| Title | 1 | Identify the study as an economic evaluation and specify the interventions being compared. | Title, Page 1 |
| **ABSTRACT** | | | |
| Abstract | 2 | Provide a structured summary that highlights context, key methods, results and alternative analyses. | Abstract, Page 1-2 |
| **INTRODUCTION** | | | |
| Background and objectives | 3 | Give the context for the study, the study question and its practical relevance for decision making in policy or practice. | Introduction, Page 2-3 |
| **METHODS** | | | |
| Health economic analysis plan | 4 | Indicate whether a health economic analysis plan was developed and where available. | Methods, First Paragraph |
| Study population | 5 | Describe characteristics of the study population (such as age range, demographics, socioeconomic, or clinical characteristics). | Methods, second Paragraph |
| Setting and location | 6 | Provide relevant contextual information that may influence findings. | Methods, Fourth Paragraph |
| Comparators | 7 | Describe the interventions or strategies being compared and why chosen. | Methods, First Paragraph |
| Perspective | 8 | State the perspective(s) adopted by the study and why chosen. | Methods, Fourth Paragraph |
| Time horizon | 9 | State the time horizon for the study and why appropriate. | Methods, First Paragraph |
| Discount rate | 10 | Report the discount rate(s) and reason chosen. | Methods, First Paragraph |
| Selection of outcomes | 11 | Describe what outcomes were used as the measure(s) of benefit(s) and harm(s). | Methods, Third Paragraph |
| Measurement of outcomes | 12 | Describe how outcomes used to capture benefit(s) and harm(s) were measured. | Methods, Third Paragraph |
| Valuation of outcomes | 13 | Describe the population and methods used to measure and value outcomes. | Methods, Third Paragraph |
| Measurement and valuation of resources and costs | 14 | Describe how costs were valued. | Methods, Fourth Paragraph |
| Currency, price date, and conversion | 15 | Report the dates of the estimated resource quantities and unit costs, plus the currency and year of conversion. | Methods, Fourth Paragraph |
| Rationale and description of model | 16 | If modelling is used, describe in detail and why used. Report if the model is publicly available and where it can be accessed. | Methods, First and second Paragraph |
| Analytics and assumptions | 17 | Describe any methods for analysing or statistically transforming data, any extrapolation methods, and approaches for validating any model used. | Methods, second Paragraph |
| Characterizing heterogeneity | 18 | Describe any methods used for estimating how the results of the study vary for sub-groups. | Methods, Last paragraph |
| Characterizing distributional effects | 19 | Describe how impacts are distributed across different individuals or adjustments made to reflect priority populations. | Methods, Fifth paragraph and Table 1 |
| Characterizing uncertainty | 20 | Describe methods to characterize any sources of uncertainty in the analysis. | Methods, Fifth paragraph and Table 1 |
| Approach to engagement with patients and others affected by the study | 21 | Describe any approaches to engage patients or service recipients, the general public, communities, or stakeholders (e.g., clinicians or payers) in the design of the study. | Not applicable |
| **RESULTS** | | | |
| Study parameters | 22 | 22 Report all analytic inputs (e.g., values, ranges, references) including uncertainty or distributional assumptions. | Table 1 |
| Summary of main results | 23 | Report the mean values for the main categories of costs and outcomes of interest and summarise them in the most appropriate overall measure. | Results, First paragraph and Table 2 |
| Effect of uncertainty | 24 | Describe how uncertainty about analytic judgments, inputs, or projections affect findings. Report the effect of choice of discount rate and time horizon, if applicable. | Results, Second paragraph |
| Effect of engagement with patients and others affected by the study | 25 | Report on any difference patient/service recipient, general public, community, or stakeholder involvement made to the approach or findings of the study | Not reported |
| **DISCUSSION** | | | |
| Study findings, limitations, generalizability, and current knowledge | 26 | Report key findings, limitations, ethical or equity considerations not captured, and how these could impact patients, policy, or practice. | Discussion |
| **OTHER RELEVANT INFORMATION** | | | |
| Source of funding | 27 | Describe how the study was funded and any role of the funder in the identification, design, conduct, and reporting of the analysis | End of manuscript |
| Conflicts of interest | 28 | Report authors conflicts of interest according to journal or International Committee of Medical Journal Editors requirement | End of manuscript |

**Tab.S7** Summary of annual survival probabilities in the partitional survival model

| State | Start month | End month | TPC | | SG | |
| --- | --- | --- | --- | --- | --- | --- |
|  |  |  | Start probability | End probability | Start probability | End probability |
| Summary | 0 | 12 | 1.000 | 1.000 | 1.000 | 1.000 |
| PFS | 0 | 12 | 1.000 | 0.058 | 1.000 | 0.162 |
| PD | 0 | 12 | 0.000 | 0.172 | 0.000 | 0.328 |
| Dead | 0 | 12 | 0.000 | 0.770 | 0.000 | 0.510 |
| Summary | 12 | 24 | 1.000 | 1.000 | 1.000 | 1.000 |
| PFS | 12 | 24 | 0.058 | 0.012 | 0.162 | 0.065 |
| PD | 12 | 24 | 0.172 | 0.058 | 0.328 | 0.130 |
| Dead | 12 | 24 | 0.770 | 0.930 | 0.510 | 0.804 |
| Summary | 24 | 36 | 1.000 | 1.000 | 1.000 | 1.000 |
| PFS | 24 | 36 | 0.012 | 0.005 | 0.065 | 0.027 |
| PD | 24 | 36 | 0.058 | 0.000 | 0.130 | 0.036 |
| Dead | 24 | 36 | 0.930 | 0.995 | 0.804 | 0.938 |
| Summary | 36 | 48 | 1.000 | 1.000 | 1.000 | 1.000 |
| PFS | 36 | 48 | 0.005 | 0.001 | 0.027 | 0.013 |
| PD | 36 | 48 | 0.000 | 0.000 | 0.036 | 0.006 |
| Dead | 36 | 48 | 0.995 | 0.999 | 0.938 | 0.981 |
| Summary | 48 | 60 | 1.000 | 1.000 | 1.000 | 1.000 |
| PFS | 48 | 60 | 0.001 | 0.000 | 0.013 | 0.006 |
| PD | 48 | 60 | 0.000 | 0.000 | 0.006 | 0.000 |
| Dead* | **48** | **60** | **0.999** | **1.000** | 0.981 | 0.994 |
| Summary | 60 | 72 | 1.000 | 1.000 | 1.000 | 1.000 |
| PFS | 60 | 72 | 0.000 | 0.000 | 0.006 | 0.002 |
| PD | 60 | 72 | 0.000 | 0.000 | 0.000 | 0.000 |
| Dead | 60 | 72 | 1.000 | 1.000 | 0.994 | 0.998 |
| Summary | 72 | 84 | 1.000 | 1.000 | 1.000 | 1.000 |
| PFS | 72 | 84 | 0.000 | 0.000 | 0.002 | 0.000 |
| PD | 72 | 84 | 0.000 | 0.000 | 0.000 | 0.000 |
| Dead# | **72** | **84** | 1.000 | 1.000 | **0.998** | **1.000** |
| Summary | 84 | 96 | 1.000 | 1.000 | 1.000 | 1.000 |
| PFS | 84 | 96 | 0.000 | 0.000 | 0.000 | 0.000 |
| PD | 84 | 96 | 0.000 | 0.000 | 0.000 | 0.000 |
| Dead | 84 | 96 | 1.000 | 1.000 | 1.000 | 1.000 |
| Summary | 96 | 108 | 1.000 | 1.000 | 1.000 | 1.000 |
| PFS | 96 | 108 | 0.000 | 0.000 | 0.000 | 0.000 |
| PD | 96 | 108 | 0.000 | 0.000 | 0.000 | 0.000 |
| Dead | 96 | 108 | 1.000 | 1.000 | 1.000 | 1.000 |
| Summary | 108 | 120 | 1.000 | 1.000 | 1.000 | 1.000 |
| PFS | 108 | 120 | 0.000 | 0.000 | 0.000 | 0.000 |
| PD | 108 | 120 | 0.000 | 0.000 | 0.000 | 0.000 |
| Dead | 108 | 120 | 1.000 | 1.000 | 1.000 | 1.000 |

*Data in yellow indicated that all patients in the TPC group entered into ‘dead’ state at the end of 60th month.

# Data in green indicated that all patients in the SG group died at the end of 84th month
